# Multi-Omics Analysis Reveals New Insights into Yak Lung Under High-Altitude Adaptation

**DOI:** 10.3390/ani16121775

**Published:** 2026-06-08

**Authors:** Ping Chen, Jian Zhang

**Affiliations:** Animal Husbandry, Pasture and Green Agricultural Institute, Gansu Academy of Agricultural Sciences, Lanzhou 730070, China; gaasxcschenping@163.com

**Keywords:** yak, lung, transcriptome, proteomics, untargeted metabolomics

## Abstract

The yak lung functions as a vital adaptive organ in cold, low-oxygen environments. Hypoxia can induce pathological remodeling in yak lung tissue, so we need to understand the molecular mechanisms of this remodeling. For this study, we used cross-omics comparative approaches, including transcriptomics, label-free proteomics, and untargeted metabolomics, to examine both normal and diseased yak lung tissues. From histological observations, the disease phenotype was identified as pulmonary emphysema. Our results showed a significant up-regulation of differentially expressed genes such as MTTP, CXCL8, RETN, and NNAT, while genes like SLC45A1, IL10, SDSL, and COL12A1 were clearly down-regulated. In the differential protein analysis, proteins such as RASSF4, EDC4, CTSC, and FECH were notably up-regulated, whereas CYP27A1, FKBP9, RAD23A, and PLSCR2 were significantly down-regulated. Metabolomic profiling revealed that palmitoyl-L-carnitine, decanoyl-L-carnitine, and o-acetylcarnitine were significantly higher in emphysematous lung tissue, whereas racemethionine and L-methionine S-oxide were significantly much lower. Also, when we compared of bulk RNA-seq, label-free proteomics, and untargeted metabolomics data revealed enrichment in three common pathways: the asthma pathway, the linoleic acid metabolism pathway, and the gastric acid secretion pathway. Of note, histamine levels were higher in both the asthma and gastric acid secretion pathways. While the mRNA expression level of BoLA-DQB was increased in the asthma pathway, its protein expression level was decreased. This study offers some initial cross-omics evidence about what happens. These findings give us a scientific basis for developing effective prevention and control strategies, which in turn can help the protection of yak health and the sustainable development of plateau animal husbandry.

## Simple Summary

Yaks live in high, cold, and low-oxygen environments, so their lungs are really important for survival. Lung diseases such as emphysema can seriously impair breathing and harm the animal’s health. In this study, the researchers compared lung tissue from healthy yaks and those with emphysema used advanced technologies to measure changes in genes, proteins, and small molecules. They found that inflammation-related substances, for example histamine were higher in diseased lungs, while several protective molecules were lower. Pathways involved in immunity and fat metabolism also changed. These findings help us understand what goes wrong in the yak lung during emphysema. By providing new insights into how lung disease develops in plateau animals, this work could help future efforts to diagnose, prevent, and treat respiratory problems in yaks. That would ultimately contribute to better animal health and more sustainable farming on the Tibetan Plateau.

## 1. Introduction

The yak, a large mammal lives only on the Qinghai–Tibet Plateau, has developed unique physiological adaptations to survive in extreme hypoxic and cold environments [1]. The lung, being a key organ for breathing and immunity, supports this high-altitude adaptation [2]. During critical stages of lung development, the pulmonary arteries and their branches undergo remodeling, resulting in the thickening of the pulmonary artery media. At the same time, changes happen in the mitochondria, cell membranes, and collagen fibers of pulmonary cells. The unique evolutionary trajectory and genetic mechanisms of the yak lung are essential for altitude adaptation [3], pointing to an urgent need to investigate these mechanisms.

Bovine respiratory disease (BRD) is a major problem for livestock health and the global economy, has its complex etiology and is hard to diagnose and manage. Thoracic ultrasonography has demonstrated strong diagnostic potential for detecting interstitial pneumonia in feedlot cattle, providing a reliable, non-invasive tool for clinical screening [4]. When looking at disease mechanisms, proteomic studies have clarified key molecular network changes triggered by *Mycoplasma bovis* infection in embryonic bovine lung cells [5]. At the same time, comprehensive prevention and control strategies for bacterial BRD remain a critical focus of current research [6]. Lung disease significantly contributes to the progressive decline in pulmonary function in yaks, leading to impaired ventilation and gas exchange, hypoxia, and carbon dioxide retention. All of these seriously hurt their health and productivity. Hypoxia is known to a direct cause of lung tissue damage [7]. Yaks living in high-altitude environments are also prone to lung emphysema, which can lead to reduced respiratory function and potentially progress to respiratory failure. Consequently, there is an urgent need to elucidate the underlying mechanisms of lung disease in yaks.

So far, transcriptomic comparative analyses have been done on the pulmonary tissues of yaks of different ages [8]. These studies showed that immune function genes were more active at 6 months of age and less so at 90 months, compared with those at 30 and 60 months. Additionally, a comparative analysis of the transcriptional profiles between yak and yellow cattle lungs identified 39 strongly positively selected genes in yak lungs, thereby providing a foundational basis for investigating the unique evolutionary processes and genetic mechanisms underlying altitude hypoxia adaptation in yaks [9]. Beyond looking at tissues, transcriptomic analyses of pulmonary artery smooth muscle cells from both yak and yellow cattle, exposed to varying oxygen concentration gradients, have been conducted [10]. This work found involvement of the HIF-1 signaling pathways and glucose metabolism pathways, along with related factors (HK2, PGK1, ENO1, ENO3, ALDOC, ALDOA), suggesting that these molecular control mechanisms may play a significant role in the adaptation of yaks to high-altitude, low-oxygen environments [10]. Earlier studies have looked at the genetic and pathway mechanisms associated with high-altitude adaptation through comparative analyses of yaks and yellow cattle [11]. But even with these advances, lung function in yaks can still be seriously harmed under certain conditions, highlighting the need for comprehensive high-throughput sequencing studies of the yak lung.

Transcriptomics, label-free proteomics, and untargeted metabolomics have become key methods for understanding molecular regulatory mechanisms and disease pathogenesis in organisms [12]. RNA sequencing of lung tissue following cigarette smoke exposure in mice has demonstrated that aerobic training can enhance lung function in cases of chronic obstructive pulmonary disease [13]. A combined proteomic and metabolomic analysis of the lung found major changes in proteins, such as adenylate cyclase 4 (ADCY4), and metabolites, including L-glutamine, guanosine monophosphate (GMP), adenosine, and guanosine. This suggests that purine metabolism may be involved in the development of lung tumor inflammation [14]. Previous research on yak lung sequencing has mostly relied on single transcriptomics [9], highlighting a gap in multi-omics approaches to study the genes, proteins, and metabolites in the lung.

Environmental factors, including hypoxia and cold temperatures at high altitudes, significantly influence the normal physiology of the yak lung [15]. We hypothesized that emphysematous yak lung shows coordinated immune and metabolic reprogramming. This study employed transcriptomics, proteomics, and untargeted metabolomics to carry out descriptive comparative analyses of healthy and diseased yak lung tissues. To our knowledge, this is the first multi-omics comparison focused specifically on yak pulmonary emphysema. The findings of this research will offer foundational data for investigating the pathogenesis and pathological alterations in yak lungs, thus offering a crucial theoretical framework for animal health management and disease prevention and control in plateau regions.

## 2. Materials and Methods

### 2.1. Experimental Animals

Yak samples were collected in February 2024 at the Qingyuan Slaughterhouse (Linxia, China). A cohort of 12 adult yaks from the same pasture (altitude: 3200 m) was randomly selected. All animals grazed together under identical management conditions, ensuring comparable nutritional status. All yaks were adult animals of similar age (approximately 3–5 years) and comparable body weight (280–320 kg). Sex distribution was not individually recorded because the slaughterhouse provides mixed-sex animals from the same herd without sex labeling; however, both groups were randomly selected from the same batch to minimize sex-related bias. All 12 yaks included in this study were slaughtered on the same date (15 February 2024) at the Qingyuan Slaughterhouse. This was deliberately planned to minimize batch effects and eliminate variations that could arise from different slaughter dates, including differences in environmental conditions, transport stress, or sample processing procedures. Prior to slaughter, all yaks underwent clinical evaluation by a trained veterinarian. Animals assigned to the experimental group (EG, experimental group) met at least three of the following criteria: persistent coughing and/or nasal discharge; tachypnea with pronounced abdominal breathing; fever (rectal temperature > 39.5 °C); and reduced appetite with lethargy. Control group (CG, control group) yaks exhibited none of these signs, showing normal respiration, absence of coughing/nasal discharge, normothermia (≤39.5 °C), normal appetite, and an alert mental state. Final group allocation was confirmed by post-mortem examination. Immediately after exsanguination, the entire respiratory tract was examined. Lungs were classified as lesioned upon observation of gross pathological lesions (pulmonary consolidation/scarring, pleural adhesions, abscesses, or pronounced atelectasis). Lungs were categorized as normal only when exhibiting a healthy appearance, uniform color, normal elasticity, and no macroscopic lesions. Following these criteria, six yaks with clear emphysematous lesions were assigned to EG and six with unequivocally healthy lungs to CG. In this exploratory study, emphysema severity was not quantitatively graded (e.g., by mean linear intercept or alveolar wall thickness); instead, a binary classification (unequivocally normal vs. clearly emphysematous) was adopted based on histopathological confirmation (Supplementary Figure S1). Euthanasia was performed via intravenous injection of pentobarbital sodium (200 mg/kg). Lung tissues for multi-omics analyses were collected within one-minute post-slaughter, rinsed with physiological saline, and immediately frozen in liquid nitrogen. All lung tissue samples were obtained from the right caudal lobe, a region consistently used for histology and omics analyses. Histopathological assessments were conducted by examiners blind to group assignment. (emphysema vs. control). All samples were stored at −80 °C until analysis. The reference genome used was Bos_mutus.BosGru_v2.0.dna.toplevel.fa (https://ftp.ensembl.org/pub/release-111, accessed on 9 July 2024).

### 2.2. Transcriptome Sequencing

Total RNA was extracted from yak lung tissue samples with TRIzol reagent (Invitrogen, Carlsbad, CA, USA) according to the manufacturer’s instructions. RNA concentration and purity were determined via a NanoDrop One spectrophotometer (Thermo Fisher Scientific, Waltham, MA, USA), with acceptable samples exhibiting A260/A280 ratios between 1.8 and 2.0 and A260/A230 ratios above 2.0. RNA integrity was further verified with an Agilent 2100 Bioanalyzer using the RNA Nano 6000 assay kit; only those samples having an RIN of 7.0 or higher proceeded to library preparation. Subsequently, first-strand cDNA was synthesized from 1 µg of total RNA using the PrimeScript RT reagent kit with gDNA Eraser (TaKaRa, Kusatsu, Japan), followed by two rounds of amplification with KAPA HiFi HotStart ReadyMix (Roche, Basel, Switzerland) under the following PCR conditions: initial denaturation at 95 °C for 3 min; 15 cycles of 98 °C for 20 s, 65 °C for 30 s, and 72 °C for 40 s; final extension at 72 °C for 5 min. An Agilent 2100 Bioanalyzer (Agilent Technologies, Santa Clara, CA, USA) was used to evaluate RNA integrity. Only samples with an RNA integrity number (RIN) ≥ 7.0, 28S/18S ratio ≥ 1.0, and A260/A280 ratio between 1.8 and 2.1 were considered acceptable. All 12 RNA samples (6 from controls, 6 from emphysema cases) met these criteria, and none were excluded from subsequent library preparation and sequencing.

For library construction, the amplified cDNA was fragmented to an average size of approximately 350 bp using a Covaris S220 focused-ultrasonicator (Covaris, Woburn, MA, USA). The NEBNext Ultra II DNA Library Prep Kit for Illumina (NEB, Ipswich, MA, USA) was used for end repair, A-tailing, and adapter ligation. Adapter-ligated fragments were purified with AMPure XP beads (Beckman Coulter, Brea, CA, USA) and then enriched by 10 cycles of PCR. The final libraries were quantified with a Qubit 4.0 fluorometer (Thermo Fisher Scientific) and assessed for size distribution on an Agilent 2100 Bioanalyzer. Sequencing was performed on an Illumina NovaSeq 6000 platform (Illumina, San Diego, CA, USA) in paired-end 150 bp mode, generating an average of 40 million raw reads per sample. Raw reads were quality-trimmed and filtered using fastp (v0.23.2) with default parameters [16]. Raw reads were processed using fastp (version 0.23.2) with the following parameters: minimum read length 50 bp, sliding window (5 bp) average quality threshold Q20, and maximum allowed N bases per read = 5. Reads containing adapter sequences were automatically trimmed. All other parameters were kept as default. Clean reads were aligned to the *Bos mutus* reference genome (BosGru_v2.0) using HISAT2 (v2.2.1) with the “--dta” parameter. Transcript quantification and read counting were performed with featureCounts (v2.0.3). Differential expression analysis was conducted using DESeq2 (v1.38.3) in R (v4.2.0). Raw read counts were normalized using the median-of-ratios method implemented in DESeq2, which accounts for differences in sequencing depth and RNA composition across samples. For differential expression analysis, DESeq2 employs a negative binomial generalized linear model. The resulting *p*-values were adjusted for multiple testing using the Benjamini–Hochberg procedure to control the false discovery rate (FDR). Genes with an adjusted *p*-value (*p*adj) < 0.05 and |log_2_FoldChange| > 1 were considered significantly differentially expressed. Functional enrichment analysis of Gene Ontology (GO) terms and Kyoto Encyclopedia of Genes and Genomes (KEGG) pathways was carried out using the clusterProfiler package (v4.6.2). Multiple testing correction was applied using the Benjamini–Hochberg method, and adjusted *p*-values < 0.05 were considered significant for enriched terms. Post hoc statistical power analysis was performed using the pwr package (v1.3-0) in R. For each significantly changed molecule, power was calculated assuming a two-sided *t*-test with α = 0.05, using the observed effect size (log_2_FC) and the pooled standard deviation. Median power values for each omics dataset are reported in Supplementary Table S1.

### 2.3. Label-Free Proteomics Sequencing

Protein was extracted from approximately 50 mg of snap-frozen normal and emphysematous yak lung tissues collected from the right caudal lobe (same as for transcriptomics) by homogenization in 500 µL of SDT lysis buffer (4% SDS, 100 mM Tris-HCl, 1 mM DTT, pH 7.6) using a Precellys Evolution homogenizer (Bertin Technologies, Montigny-le-Bretonneux, France) at 6500 rpm for three cycles of 30 s each, with 30 s intervals on ice. The homogenate was boiled at 95 °C for 10 min, then centrifuged at 14,000× *g* for 40 min at 4 °C. The supernatant containing the total protein was collected, and its concentration was determined using a BCA protein assay kit (Thermo Fisher Scientific, Waltham, MA, USA) with bovine serum albumin as the standard. For each sample, 100 µg of protein was taken for downstream processing. Disulfide bonds were reduced by adding dithiothreitol (DTT) to a final concentration of 5 mM and incubating at 37 °C for 1 h. Subsequently, cysteine residues were alkylated by adding iodoacetamide (IAA) to a final concentration of 10 mM and incubating in the dark at room temperature for 45 min. The alkylation reaction was quenched by adding an additional 5 mM DTT. Proteins were then precipitated using cold acetone (1:4 *v*/*v*) at −20 °C overnight. The precipitate was washed twice with cold 80% acetone, air-dried, and redissolved in 50 µL of 25 mM ammonium bicarbonate (ABC) buffer. Trypsin (Sequencing Grade Modified Trypsin, Promega, Madison, WI, USA) was added at an enzyme-to-protein ratio of 1:50 (*w*/*w*), and the digestion was carried out at 37 °C for 16 h. The reaction was stopped by acidifying the mixture with formic acid to a final concentration of 1% (pH < 3).

The resulting peptide mixture was desalted using a C18 StageTip (Thermo Fisher Scientific, Waltham, MA, USA). The tip was first activated with 100% acetonitrile (ACN), equilibrated with 0.1% formic acid (FA) in water, loaded with the acidified sample, washed with 0.1% FA to remove salts and impurities, and finally eluted with 70% ACN containing 0.1% FA. The eluted peptides were concentrated in a vacuum centrifuge and reconstituted in 20 µL of 0.1% FA for LC-MS/MS analysis. To monitor instrument stability and reproducibility, a pooled quality control (QC) sample (prepared by mixing equal aliquots of all study samples) was injected every 10 injections throughout the analytical run. QC samples were also analyzed at the beginning and end of each batch. The coefficient of variation (CV) of feature intensities across QC samples was calculated, and only features with CV < 30% in QCs were retained for downstream analysis. The analysis was performed on a Q Exactive HF-X Hybrid Quadrupole-Orbitrap mass spectrometer (Thermo Fisher Scientific, Waltham, MA, USA) coupled to an EASY-nLC 1200 system (Thermo Fisher Scientific, Waltham, MA, USA). Peptides were separated on a 50 cm analytical column (PepMap RSLC C18, 2 µm, 100 Å) using a 120 min gradient from 4% to 28% mobile phase B (80% ACN, 0.1% FA) at a flow rate of 300 nL/min. Data-dependent acquisition (DDA) mode was used for operating the mass spectrometer. Orbitrap-based full MS scans were collected at a resolution of 120,000 (at *m*/*z* 200) over a range of 350–1500 *m*/*z*, with an automatic gain control (AGC) target of 3 × 10^6^. The top 20 most intense precursors were selected for higher-energy collisional dissociation (HCD) fragmentation with a normalized collision energy of 28%, and the MS/MS spectra were acquired at a resolution of 15,000. Raw data files were processed using MaxQuant software (version 2.0.3.0) against the *Bos mutus* grunniens reference proteome database (UniProt). The search included variable modifications of methionine oxidation and fixed modification of carbamidomethylation on cysteine. The false discovery rate (FDR) was set to 1% at both the peptide and protein levels. Protein intensities were normalized using the MaxLFQ algorithm within MaxQuant software, which enables accurate label-free quantification by considering only common peptides between runs. Differential expression analysis was performed in Perseus software (version 1.6.15.0) using a two-sample *t*-test. Given the exploratory nature of this study and the relatively small sample size (*n* = 6 per group), we prioritized the identification of candidate proteins with consistent expression changes while controlling the identification FDR at 1% at the peptide and protein levels via MaxQuant. To avoid over-correction that could mask biologically relevant signals in this discovery-phase analysis, we did not apply an additional FDR adjustment to the *t*-test *p*-values. All reported differentially expressed proteins should be considered as candidates requiring validation in future studies. Differentially expressed proteins (DEPs) were identified with filtering criteria of |Log_2_(Fold Change)| > 0.585 (equivalent to a 1.5-fold change) and a *p*-value < 0.05 (derived from a two-sample *t*-test). Functional enrichment analysis of Gene Ontology (GO) terms and Kyoto Encyclopedia of Genes and Genomes (KEGG) pathways for the DEPs was performed using the clusterProfiler R package (version 4.6.2), with significance determined by Fisher’s exact test (adjusted *p*-value < 0.05). Protein–protein interaction networks were predicted using the STRING database (version 11.5) with a confidence score threshold of 0.7.

### 2.4. Untargeted Metabolomics Sequencing

For untargeted metabolomics analysis, approximately 30 mg of snap-frozen yak lung tissue was accurately weighed and transferred into a pre-chilled 2 mL microcentrifuge tube. To each sample, 1000 µL of ice-cold extraction solvent consisting of methanol:chloroform:water (75:25:1, *v*/*v*/*v*) was added along with two stainless steel grinding beads (3 mm). Tissue homogenization was performed using a TissueLyser II (Qiagen, Hilden, Germany) at 50 Hz for 60 s, repeated for two cycles with a 30 s interval on ice to prevent overheating. The homogenate was then subjected to ultrasonication in a water bath at 25 °C for 30 min, followed by incubation on ice for an additional 30 min to facilitate complete metabolite extraction. The sample mixture underwent centrifugation at 12,000× *g* for 10 min under 4 °C. All of the supernatant was then gently moved to a new 2 mL tube and concentrated to dryness using a vacuum concentrator (Eppendorf Concentrator Plus, Eppendorf, Hamburg, Germany) at 45 °C. The dried metabolite extract was subsequently reconstituted in 200 µL of a 50% acetonitrile aqueous solution containing 4 ppm 2-chloro-L-phenylalanine as an internal standard for quality control and signal normalization. Peak areas were normalized to this internal standard to correct for technical variations. The final solution was vortexed thoroughly, centrifuged, and the supernatant was filtered through a 0.22 μm PTFE membrane filter into a glass vial insert placed within an LC-MS autosampler vial for analysis.

Liquid chromatography–mass spectrometry analysis was performed on an Agilent 1290 Infinity II UHPLC system (Agilent Technologies, Santa Clara, CA, USA) coupled to an Agilent 6470 Triple Quadrupole LC/MS system (Agilent Technologies, Santa Clara, CA, USA). Chromatographic separation was achieved on an ACQUITY UPLC HSS T3 column (Waters, Milford, MA, USA) with dimensions 2.1 mm × 100 mm, and particle size 1.8 µm, maintained at 45 °C. The mobile phase consisted of (A) 0.1% formic acid in water and (B) 0.1% formic acid in acetonitrile. The gradient elution program was as follows: 0–2 min, 2% B; 2–10 min, 2–50% B; 10–14 min, 50–98% B; 14–16 min, 98% B; 16–16.1 min, 98–2% B; 16.1–20 min, 2% B for re-equilibration. The flow rate was 0.3 mL/min and the injection volume was 2 µL. The mass spectrometer was operated in dynamic multiple reaction monitoring (dMRM) mode with both positive and negative electrospray ionization (ESI). Source parameters were set as follows: gas temperature 325 °C, gas flow 10 L/min, nebulizer pressure 35 psi, sheath gas temperature 375 °C, sheath gas flow 12 L/min, capillary voltage 3500 V (positive) and 3000 V (negative). Data acquisition and processing were conducted using Agilent MassHunter Workstation Software (version 10.1Agilent Technologies, Santa Clara, CA, USA). Metabolite peaks were integrated, aligned, and quantified relative to the internal standard. Univariate and multivariate statistical analyses were performed using MetaboAnalyst 5.0 (web-based platform, https://www.metaboanalyst.ca, accessed on 9 July 2024). Data were subjected to Pareto scaling prior to multivariate analysis. Orthogonal partial least squares-discriminant analysis (OPLS-DA) was performed to maximize class separation. The model performance was evaluated using the goodness-of-fit (R^2^) and predictive ability (Q^2^) metrics obtained from seven-fold cross-validation. To assess the risk of overfitting, permutation tests (*n* = 200) were conducted, and the R^2^ and Q^2^ values of the permuted models were compared with those of the original model. Differentially expressed metabolites (DEMs) were identified based on a variable importance in projection (VIP) score > 1.0 from the orthogonal partial least squares-discriminant analysis (OPLS-DA) model and a *p*-value< 0.05 from Student’s *t*-test. For the OPLS-DA models, the following validation metrics were obtained: in positive ion mode, R^2^ = 0.99, Q^2^ = 0.91; in negative ion mode, R^2^ = 0.99, Q^2^ = 0.94. Permutation tests (*n* = 200) confirmed the robustness of the models, with no overfitting observed. The identified DEMs were subsequently subjected to pathway enrichment analysis and topological analysis within MetaboAnalyst, and the results were mapped to the KEGG pathway database for systemic functional interpretation.

### 2.5. Comparative Multi-Omics Pathway Analysis

For the integrated pathway analysis, we employed a descriptive overlap approach rather than a statistical meta-analysis with combined *p*-values. Redundant KEGG terms were merged using clusterProfiler’s simplify function (cutoff = 0.7, Jaccard similarity), retaining the most significant term per cluster, and parent–child relationships were manually checked to avoid overcounting. The process was as follows: Lists of significantly changed mRNAs (*p*adj < 0.05, |log_2_FC| > 1), proteins (*p* < 0.05, |log_2_FC| > 0.585), and metabolites (VIP > 1.0, *p* < 0.05) were independently mapped to KEGG pathways using the clusterProfiler package (v4.6.2) for transcriptomic and proteomic data, and MetaboAnalyst 5.0 for metabolomic data. For each KEGG pathway, we recorded whether it contained at least one significant molecule from each omics dataset. Pathways were classified as: common pathways (triple overlap) containing at least one significant molecule from all three omics datasets; paired overlaps containing significant molecules from two of the three datasets; and dataset-specific pathways containing significant molecules from only one dataset.

## 3. Results

### 3.1. Histological Observation of Yak Lung

The normal lung tissue of yaks (Supplementary Figure S1A) exhibits intact structural integrity, with clear and regularly shaped alveolar contours, uniformly thin alveolar walls, evenly sized alveolar spaces, no proliferation of pulmonary interstitium or infiltration of inflammatory cells, well-defined structural layers of bronchi and alveolar ducts at all levels, and no evident pathological changes. In contrast, the emphysematous lung tissue (Supplementary Figure S1B) displays typical pathological features, including the obvious enlargement of multiple alveolar spaces, the rupture of partial alveolar walls, and the fusion of alveoli to form larger cystic cavities, which is a classic histological manifestation of pulmonary emphysema; concomitantly, the alveolar walls appear thin and disrupted due to alveolar dilation; furthermore, extensive infiltration of inflammatory cells, primarily leukocytes such as lymphocytes and neutrophils, is observed in the alveolar walls and surrounding tissues, indicating the presence of an inflammatory response.

### 3.2. Transcriptomic Analysis

**Figure 1:**
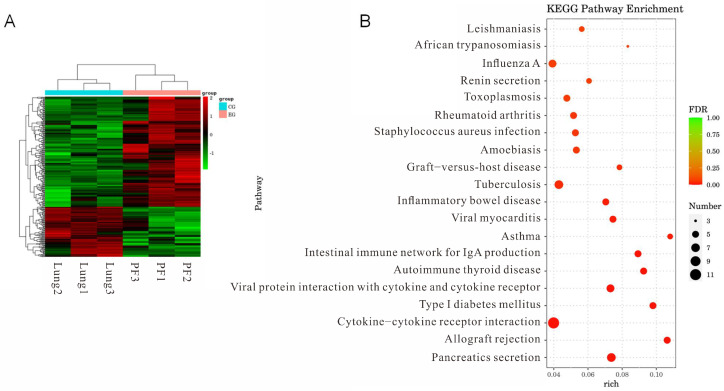
Differential genes in yak lung transcriptome analysis. (**A**) Heatmaps of transcriptomics; (**B**) KEGG analysis of transcriptomics.

In this study, lung tissue was divided into normal lung tissue and emphysematous lung tissue, with normal lung tissue serving as the control group (CG) and emphysematous lung tissue as the experimental group (EG). The transcriptomic comparison between normal and emphysematous yak lung identified 254 differentially expressed genes, including 174 up-regulated genes and 80 down-regulated genes (Table 1). *MTTP*, *CXCL8*, *RETN*, *NNAT*, *SLC5A9*, *CA2*, *PLA2G5*, *ALOX5*, *RIMS1* and *COL11A1* were significantly up-regulated differential genes. *SLC45A1*, *IL10*, *SDSL*, *COL12A1*, *SERPINB8*, *ADCYAP1*, *EIF2B2*, *ARSD*, *CPE* and *TNFRSF11B* were significantly down-regulated differential genes. All these listed differentially expressed genes are statistically significant, with an adjusted *p*-value (*p*adj) < 0.05 (Benjamini–Hochberg correction) and |log_2_FC| > 1. The screening criteria for all differential genes were |log_2_FC| > 1 and *p*-value < 0.05. Heatmap analysis demonstrated clear separation between normal and emphysematous lung samples, which could be used for subsequent enrichment analysis (Figure 1A). To further assess the clustering structure of transcriptomic data, principal component analysis (PCA) was performed on the normalized gene expression profiles. As shown in Supplementary Figure S4, PC1 and PC2 explained 59% and 15% of the total variance, respectively. Although the two groups (CG and EG) showed a trend toward separation, a partial overlap was observed, indicating that the transcriptional differences between normal and emphysematous lungs are not dominated by the first two principal components. Nevertheless, the clear separation achieved by supervised analysis (Figure 1A heatmap) and the large number of differentially expressed genes (*n* = 254) support the biological significance of the observed transcriptomic alterations. GO enrichment analysis of these DEGs showed significant enrichment in biological processes and molecular functions including MHC class II protein complex, phosphatidylcholine transporter activity, chemokine receptor binding, and chemokine activity, alongside other immune- and stimulus-related terms (Supplementary Figure S2). KEGG pathway enrichment analysis further demonstrated that these DEGs were significantly concentrated in pathways such as asthma, intestinal immune network for IgA production, autoimmune thyroid disease, type I diabetes mellitus, and allograft rejection, as well as other immune- and inflammation-related pathways (Figure 1B). Among the up-regulated genes (all with adjusted *p* < 0.05), CXCL8 (a pro-inflammatory chemokine), PLA2G5 (involved in lipid mediator production), and COL11A1 (a collagen remodeling-related gene) may contribute to emphysematous inflammation and tissue destruction. The down-regulated IL10 (adjusted *p* < 0.05) suggests impaired immune suppression, while SLC45A1 and COL12A1 (adjusted *p* < 0.05) may affect alveolar integrity.

### 3.3. Label-Free Proteomics

**Figure 2:**
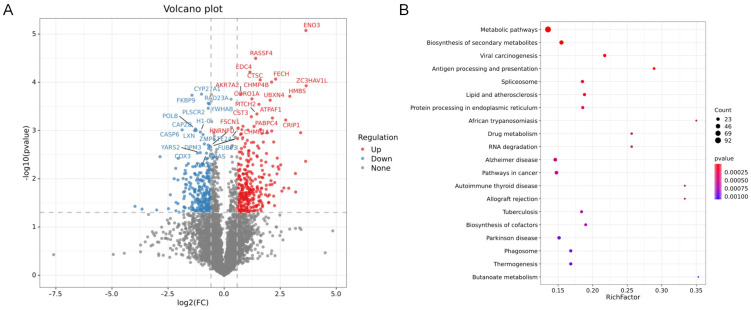
Label-free proteomic analysis of differential proteins in yak lung. (**A**) Volcano plot of label-free proteomics; (**B**) KEGG analysis of label-free proteomics.

Label-free proteomic analysis of normal and emphysematous lungs of yaks revealed a total of 554 differentially expressed proteins, including 255 down-regulated proteins and 299 up-regulated proteins (Table 2). Among these, the volcano plot (Figure 2A) illustrates the distribution of up-regulated and down-regulated proteins, highlighting significant changes between the two groups. The significantly up-regulated differential proteins included RASSF4, EDC4, CTSC, FECH, AKR7A2, CHMP4B and ZC3HAV1L, while the significantly down-regulated differential proteins included CYP27A1, FKBP9, RAD23A, PLSCR2 and YWHAB. All differentially expressed proteins satisfied |log_2_FC| > 0.585 and *p* < 0.05. Up-regulated proteins (RASSF4, EDC4, CTSC, FECH, AKR7A2, CHMP4B, ZC3HAV1L) are involved in cell cycle control, mRNA decay, matrix degradation, heme synthesis, and antiviral defense; down-regulated ones (CYP27A1, FKBP9, RAD23A, PLSCR2, YWHAB) function in cholesterol metabolism, protein folding, DNA repair, and signal transduction. Heatmap analysis of differential proteins showed that samples within the same group clustered closely, and the overall expression patterns of differential proteins exhibited good consistency with strong reproducibility, making them suitable for subsequent functional enrichment analysis (Supplementary Figure S3). To evaluate the unsupervised clustering of proteomic data, PCA was applied to the normalized protein abundance matrix (Supplementary Figure S5). The first two principal components explained 34.4% and 24.4% of the variance, respectively. The PCA score plot did not reveal a clear separation between CGs and EGs, consistent with the moderate effect sizes observed for many differentially expressed proteins. However, the robustness of the proteomic differences is supported by the large number of significantly altered proteins (*n* = 554, |log2FC| > 0.585, *p* < 0.05) and the well-clustered heatmap (Supplementary Figure S3), suggesting that the biological signal, though not captured by the first two PCs, is nonetheless present in the high-dimensional space. KEGG pathway enrichment analysis (Figure 2B) revealed that the differential proteins obtained by comparing normal and emphysematous lungs were mainly enriched in pathways such as Metabolic pathways, Biosynthesis of secondary metabolites, Viral carcinogenesis, Antigen processing and presentation, Spliceosome, Lipid and atherosclerosis, Protein processing in endoplasmic reticulum, African trypanosomiasis, Drug metabolism, RNA degradation, Alzheimer disease, Pathways in cancer, Autoimmune thyroid disease, Allograft rejection, Tuberculosis, Biosynthesis of cofactors, Parkinson disease, Phagosome, Thermogenesis, and Butanoate metabolism, among others.

### 3.4. Untargeted Metabolomics

Metabolites in normal and emphysematous lungs of yaks were classified into positive ion mode metabolites and negative ion mode metabolites by untargeted metabolomics analysis. Among them, the positive ion pattern metabolites of normal and emphysematous lungs were significantly different (OPLS-DA model, permutation test *p* < 0.01), showing clear separation between groups, PLS-DA score plot revealed that the positive ion mode metabolites of normal and emphysematous lungs were significantly different, showing clear separation between groups, with PC1 and PC2 explaining 18.9% and 16.4% of the variance, respectively (Supplementary Figure S3A). The PLS-DA permutation test diagram showed that the model was reliable, with Q^2^ reaching 0.53 (Supplementary Figure S3B). Similarly, the negative ion pattern metabolites of normal and emphysematous lungs were significantly different, as illustrated in the PLS-DA score plot (Supplementary Figure S3C), where PC1 and PC2 accounted for 17% and 7% of the variance, respectively. The PLS-DA permutation test diagram showed that the model was reliable, with R^2^ and Q^2^ values of 0.94 and 0.19, respectively (Supplementary Figure S3D). Through the above PLS-DA analysis of normal lungs and emphysematous lungs, it was found that the metabolic groups of the two groups were significantly different, and the intra-group reproducibility was good, which could be used for subsequent functional enrichment analysis.

**Figure 3:**
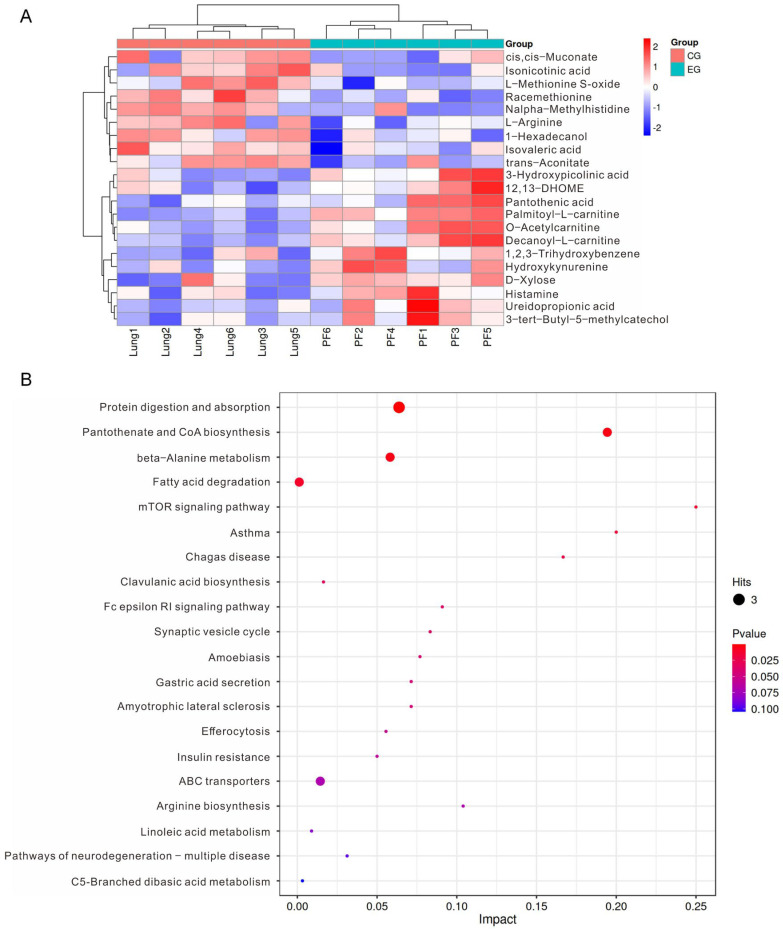
Analysis of secondary differential metabolites in untargeted metabolomics of yak lung. (**A**) Clustering heatmaps of the metabolomic differential metabolites of the whole target. (**B**) Pathway enrichment analysis of secondary differential metabolites in untargeted metabolomics. The size of each dot represents the number of hits (differential metabolites in the pathway), and the color gradient indicates the *p*-value (smaller *p*-value in red, larger in blue).

The R package pheatmap was used to scale the secondary differential metabolite matrix data, and bidirectional clustering of samples and differential metabolites was carried out. As shown in the heatmap (Figure 3A), cis, cis-muconate, isonicotinic acid, L-Methionine S-oxide, racemethionine, Nalpha-methylhistidine and L-arginine were highly expressed in emphysematous lungs compared to the control group. 1-Hexadecanol, isovaleric acid, and trans-aconitate were lowly expressed in emphysematous lungs, while 3-hydroxypropionic acid, 12,13-DHOME, pantothenic acid, palmitoyl-L-carnitine, O-acetylcarnitine, decanoyl-L-carnitine, D-xylose, histamine, ureidopropionic acid, and 3-tert-butyl-5-methyloctanol were highly expressed in emphysematous lungs. Among all the above differential metabolites, palmitoyl-L-carnitine, decanoyl-L-carnitine, and O-acetylcarnitine were the most significantly up-regulated in emphysematous lungs, as indicated by the volcano plot (Supplementary Figure S4). Racemethionine and L-methionine S-oxide were significantly down-regulated in emphysematous lungs. KEGG enrichment analysis revealed that differential metabolites were mainly enriched in pathways such as the mTOR signaling pathway, asthma pathway, protein digestion and absorption, beta-Alanine metabolism, fatty acid degradation, Chagas disease, synaptic vesicle cycle, ABC transporters, arginine biosynthesis, and linoleic acid metabolism, among others (Figure 3B).

### 3.5. Correlation Analysis of Substances with Multiple Omics Differences

Correlation analysis of differential metabolites and differential proteins in emphysematous lung showed that 12,13-DHOME was positively correlated with EIF3j, and histamine was positively correlated with ANKRD53, SLC4A1, MTTP, SHISA3, ATP6V0D2, SPDEF, ADAM28, and FILIP1. Palmitoyl-L-carnitine and pantothenic acid exhibited positive correlation with multiple proteins, including *Bclaf1*, *EIF3j*, *HMBS*, *DENND1C*, *Periaxin*, *JAM3*, *FAM21A*, *PPH* and *CRIP1*.

**Figure 4:**
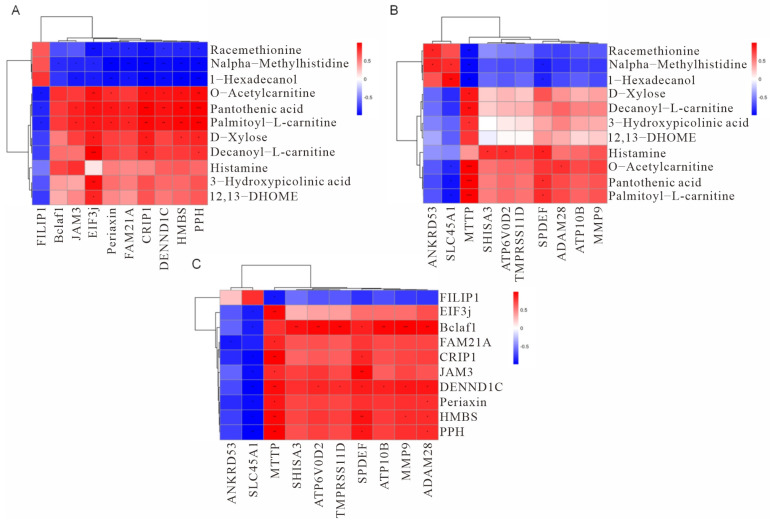
Correlation analysis of different substances in yak lung. (**A**) Correlation analysis of differential metabolites and differential proteins. (**B**) Correlation analysis of differential metabolites and differential genes. (**C**) Correlation analysis of differential proteins and differential genes. * *p* < 0.05, ** *p* < 0.01, *** *p* < 0.001.

Palmitoyl-L-carnitine and pantothenic acid were negatively correlated with *FILIP1*. Nalpha-methylhistidine and 1-hexadecanol were negatively correlated with *Bclaf1*, *EIF3j*, *HMBS*, *DENND1C*, *Periaxin*, *JAM3*, *FAM21A*, *PPH* and *CRIP1* (Figure 4A). Correlation analysis between differential metabolites and differential genes in emphysematous lung revealed that histamine was positively correlated with *SHISA3, ATP6V0D2* and *SPDEF*. *MTTP* was negatively correlated with racemethionine, Nalpha-methylhistidine and 1-hexadecanol. MTTP with histamine, 3-hydroxypicolinic acid, D-xylose, O-acetylcarnitine, pantothenic acid, decanoyl-L-carnitine, palmitoyl-L-carnitine and 12, 13-DHOME showed positive correlation regulation (Figure 4B). Emphysema of the lung protein, associated with differences in gene analysis, found that *EIF3j Bclaf1*, *FAM21A*, *CRIP1*, *JAM3*, *DENND1C*, *Periaxin*, *HMBS* and *PPH* and *MTTP SHISA3*, *ATP6V0D2*, *TMPRSS11D*, *SPDEF*, *ATP10B*, *MMP9* and *ADAM28* showed positive correlation. *FILIP1* was positively correlated with *ANKRD53* and *SLC45A1* (Figure 4C). These correlations suggest that MTTP, SPDEF, and ATP6V0D2 are positively associated with histamine-related inflammatory signaling, while FILIP1 is negatively correlated with fatty acid metabolites (palmitoyl-L-carnitine and pantothenic acid). The positive correlation between histamine and multiple genes (e.g., SHISA3, ATP6V0D2, SPDEF) may reflect in promoting airway remodeling and immune cell recruitment. Conversely, the negative correlation between MTTP and racemethionine/Nalpha-methylhistidine suggests a potential metabolic association that could influence methionine cycle and histidine metabolism during emphysema progression.

### 3.6. Joint Analysis of Multiple Omics Enrichment Pathways

**Figure 5:**
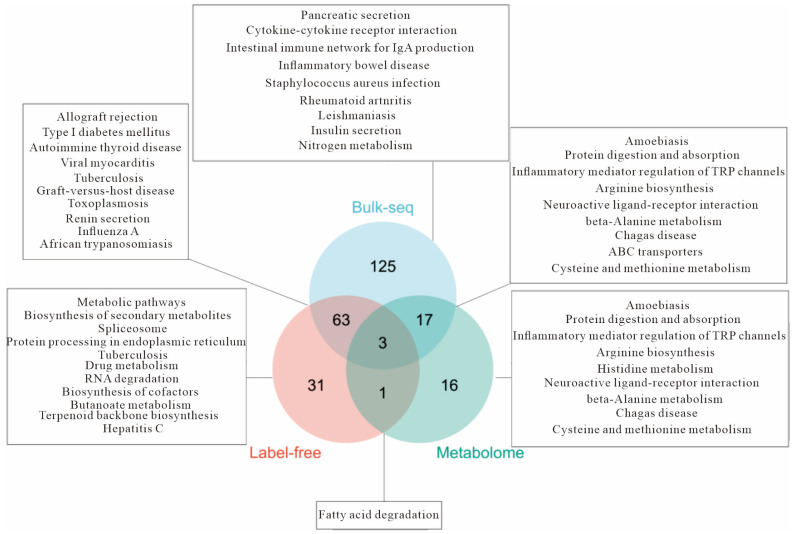
Combined analysis of multiple omics pathways in yak lung.

In this study, bulk-seq, label-free and metabolome were jointly analyzed to discover their common biological pathways. Figure 5 shows the numbers of shared and dataset-specific pathways, with 10 representative pathways listed for illustration. All enriched pathways met the significance thresholds (adjusted *p*-value < 0.05 for transcriptomics and proteomics; *p*-value < 0.05 and VIP > 1 for metabolomics). The three common pathways (asthma, linoleic acid metabolism, and gastric acid secretion) had the following adjusted *p*-values: asthma pathway (transcriptomics *p*adj = 0.012, proteomics *p*adj = 0.035, metabolomics *p* = 0.008); linoleic acid metabolism (transcriptomics *p*adj = 0.024, proteomics *p*adj = 0.041, metabolomics *p* = 0.021); gastric acid secretion (transcriptomics *p*adj = 0.018, proteomics *p*adj = 0.029, metabolomics *p* = 0.014). The detailed statistical significance, including −log_10_(*p*-values), number of significant molecules, and enrichment ratios for each omics dataset, is presented in Table 3. Following the descriptive integration approach described in Section 2.5, bulk-seq and label-free had 66 common biological pathways, bulk-seq and metabolome had 20 common biological pathways, and label-free and metabolome had four common biological pathways. There are 125 biological pathways specific to bulk-seq, 31 biological pathways specific to label-free, and 16 biological pathways specific to metabolome. Bulk-seq, label-free and metabolome were enriched into three common pathways, the asthma pathway, linoleic acid metabolism pathway, and gastric acid secretion pathway. In the asthma pathway, the mRNA levels of HLA-DQA2, BoLA-DQB, and IL10 were altered, and the protein levels of FceRI gamma, IGHM, BoLA-DRA, and BoLA-DQB were also changed. The only metabolite that changes the asthma pathway was histamine. The mRNA changes in linoleic acid metabolism pathway include *PLA2G5* and *CYP2C19*, and the protein changes in linoleic acid metabolism pathway included *PLA2* and *CYP450*. The metabolites that change the linoleic acid metabolism pathway were 12, 13-DHOME. Gastric acid secretion pathway change mRNA included *CA2, PRKCG* and *CAMK2A*. Gastric acid secretion pathway change protein included *PLCB2, CALM2, Ezrin, PLCB1* and *GNAI1*. The metabolite of the gastric acid secretion pathway change was histamine (Table 4). In summary, histamine was up-regulated in both the asthma pathway and gastric acid secretion pathway, and the mRNA level of *BoLA-DQB* in the asthma pathway was up-regulated. However, the protein level of *BoLA-DQB* was down-regulated.

**Table 3:** Three pathways showing the different proteins and metabolites involved.

Pathway	Gene	Protein	Metabolite
Asthma pathway	HLA-DQA2 BoLA-DQB IL10	FCER1G IGHM BoLA-DRA BoLA-DQB	histamine
Linoleic acid metabolism pathway	PLA2G5 CYP2C19	PLA2 CYP450	12,13-DHOME
Gastric acid secretion pathway	CA2 PRKCG CAMK2A	PLCB2 CALM2 Ezrin PLCB1 GNAI1	histamine

Red: up-regulated. Blue: down-regulated.

**Table 4:** Statistical significance of the three common KEGG pathways across transcriptomics, label-free proteomics, and untargeted metabolomics.

Pathway	Omics Dataset	Significance Score (*p*adj or *p*)	−log_10_(Significance)	Number of Significant Molecules	Enrichment Ratio (Rich Factor)
Asthma pathway (ko05310)	Transcriptomics	*p*adj = 0.012	1.92	3 genes	0.25
	Proteomics	*p*adj = 0.035	1.46	4 proteins	0.31
	Metabolomics	*p* = 0.008	2.10	1 metabolite (histamine)	–
	Average significance	–	1.83 (geometric mean)	–	–
Linoleic acid metabolism (ko00591)	Transcriptomics	*p*adj = 0.024	1.62	2 genes	0.18
	Proteomics	*p*adj = 0.041	1.39	2 proteins	0.22
	Metabolomics	*p* = 0.021	1.68	1 metabolite (12,13-DHOME)	–
	Average significance	–	1.56 (geometric mean)	–	–
Gastric acid secretion (ko04971)	Transcriptomics	*p*adj = 0.018	1.74	3 genes	0.20
	Proteomics	*p*adj = 0.029	1.54	5 proteins	0.33
	Metabolomics	*p* = 0.014	1.85	1 metabolite (histamine)	–
	Average significance	–	1.71 (geometric mean)	–	–

### 3.7. Joint Analysis of Multiple Omics Regulatory Networks

**Figure 6:**
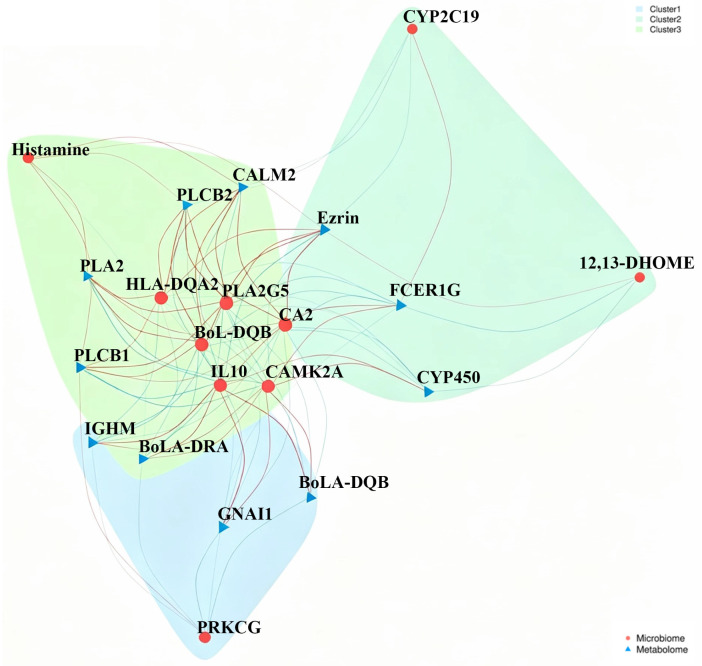
Correlation-based network of multi-omics features in yak lung. Red lines indicate positive regulation, and blue lines indicate negative regulation.

The transcriptomic, proteomic and metabolomic regulatory networks of yak lung were jointly analyzed. Selected differentially expressed mRNAs of the asthma pathway, linoleic acid metabolism pathway, and gastric acid secretion pathway are shown in Figure 6. Regulatory network analysis by differential protein and differential metabolite showed that histamine has a positive regulatory relationship with *Ezrin*, *CALM2*, *PLCB2*, *PLA2* and *PLCB1*, while histamine has a negative regulatory relationship with *BoLA-DRA*. There was a positive regulatory relationship between 12,13-DHOME and *Ezrin*, and a negative regulatory relationship between 12,13-DHOME and *FCER1G* and *CYP450*. Both histamine and 12,13-DHOME had positive regulatory relationships with *Ezrin*. CYP2C19 was positively regulated with *FCER1G*, and *CYP2C19* was negatively regulated with *Ezrin, CALM2* and *PLCB2*. There was a positive regulatory relationship between *PRKCG* and *PLCB1*, and a negative regulatory relationship between *PRKCG* and *IGHM*, *BoLA-DRA*, *GNAI1* and *BoLA-DQB*.

## 4. Discussion

Hypoxia is closely associated with pulmonary remodeling [17], and the lungs of yaks in high-altitude areas also undergo emphysematous changes, affecting their respiratory function. Lung emphysema is a complex pathological process involving the interaction of multiple cellular and molecular mechanisms [18]. In this study, bulk RNA-seq, label-free proteomics and untargeted metabolomics were used to study mRNAs, proteins and metabolites at different levels in normal and diseased yak lungs. Based on the common pathways enriched across our multi-omics data (e.g., ECM-receptor interaction, TGF-β signaling, oxidative phosphorylation, and lipid metabolism reprogramming), we propose a unifying conceptual model: a hypoxia-driven inflammation–protease–oxidative stress axis. In this cascade, hypoxia first induces transcriptional up-regulation of inflammatory cytokines (e.g., CXCL8) and matrix metalloproteinases (e.g., MMP9). This leads to a protease/anti-protease imbalance at the protein level (e.g., up-regulation of CTSC). Subsequently, metabolic reprogramming occurs, including accumulation of oxidized lipids (e.g., 12,13-DHOME) and dysregulation of energy metabolism. The final consequence is alveolar wall destruction and emphysema. In the following discussion, we first describe the correlative findings derived directly from our omics data, then discuss potential biological interpretations, clearly distinguishing observed associations from speculative mechanisms that require functional validation.

CXCL8 was a chemokine that plays an important role in participating in and regulating lung physiology and inflammation [19]. Antagonizing *CXCL8/*glycosaminoglycan binding reduces lung inflammation as well as associated lung tissue damage due to LPS and TS [20], TLR2-ROS signaling played a crucial role in the 30 kDa Ag-mediated expression of CXCL8 and CCL2 in human monocytes [21]. *PLA2G5* contributed to viral-like-induced lung inflammation through macrophage proliferation and LA/Ffar1 lung cell recruitment [22], our findings are consistent with a potential role for this protein in yak lung emphysema, but functional studies are needed to confirm this. COL11A1 was closely related to the structure and function of lung cancer [23]. Abnormal deposition of collagen was a significant feature in the process of lung remodeling [24], and the elevated COL11A1 expression observed in this study correlates with this feature. However, it is important to note that these findings are correlative, and the inferred regulatory relationships require functional validation.

In summary, although the regulatory mechanisms of *CXCL8*, *PLA2G5* and *COL11A1* in lung pathology are not fully understood, they may indirectly influence the occurrence and development of lung emphysema through their involvement in inflammation, metabolism and extracellular matrix remodeling. Future studies need to further investigate the specific mechanism of *CXCL8*, *PLA2G5* and *COL11A1* in yak lung emphysema. IL10 was an important anti-inflammatory cytokine, which can inhibit inflammation and immune response [25]. Studies with IL10-GFP mice revealed that *IL10*-expressing cells were increased after injury in mice and colocalized with moMacs [26], and our data raise the possibility that IL10 down-regulation may contribute to emphysema progression in yaks. However, validation through targeted intervention experiments is necessary. The regulatory role of *IL10* may have a potential benefit in lung disease. COL12 recombinant protein was an important connective tissue protein, abnormal deposition and remodeling of collagen was one of the key features of pulmonary remodeling. Although the direct association between *COL12A1* and lung emphysema remains to be further elucidated, the role of its encoded collagen in the pathological process cannot be ignored. *SLC45A1, SDSL, ARSD, CPE* and *TNFRSF11B* were also low expressed in yak lung emphysema, but their specific functions in vivo and their relationship with lung emphysema are still unclear. However, these genes and proteins may indirectly participate in the occurrence and development of lung emphysema by affecting cell proliferation, apoptosis, metabolism and other processes. More work will be needed to further explore what these genes do in lung emphysema and look for potential therapeutic targets.

The label-free proteomics technique allows for direct qualitative and quantitative analysis of proteins without the need to label samples [27], which has a unique advantage in the study of yak lung emphysema because it can avoid the errors and interference that may be introduced by the labeling process. CTSC expression and secretion were associated with lung metastasis in human breast tumors [28]. CTSC, as an enzyme capable of degrading extracellular matrix [29], is observed to be up-regulated in emphysematous yak lung tissue. One might guess—it is tempting to speculate that CTSC might counteract excessive matrix deposition. The high expression of CTSC in emphysematous yak lung tissue could be interpreted as a possible compensatory response; however, this interpretation remains speculative without direct functional evidence, and its precise role requires further investigation. The specific mechanism of action of CTSC in lung remains to be further studied. Based on its potential role in this condition, the development of targeted therapeutic drugs for CTSC offers new ideas and methods for treating lung. CHMP4B and ZC3HAV1L proteins were up-regulated in yak emphysematous lung tissue. Although the direct relationship between these two genes and the lungs was unknown, CHMP4B and ZC3HAV1L may indirectly affect the occurrence or development of lung through some biological processes that they participate in. CYP27A1 has been identified as a diagnostic marker for the prognosis and immunity in lung adenocarcinoma, and the expression of CYP27A1 was positively correlated with the infiltration level of most immune cells [30]. Our result matches what Qianjun Zhu et al. found [31]. In our study, CYP27A1 was down-regulated in emphysematous yak lung tissue, which might link it to the disease process. Still, what exactly CYP27A1 does in yak lung emphysema remains unclear, so we should be careful about applying findings from other studies.

When tissue gets damaged or inflamed, histamine gets released. This makes capillaries and venules more permeable and leads to local tissue edema. In our study, histamine levels were higher in emphysematous lung than that in normal lung. This observation suggests a correlative association between histamine elevation and the emphysematous process in yak lungs. It’s worth stressing that these results are only correlative, so functional studies are needed to figure out cause and effect. Elevated levels of five fatty acid metabolites (8-HETE, 12, 13-DHOME, 13-HODE, 9-HODE, and 9,12, 13-THOME) increased the risk of ovarian cancer [32]. Among them, 12,13-DHOME was an endogenous bioactive lipid, which played an important physiological role in human body. In addition to the 12, 13-DHOME study in ovarian cancer, the untargeted metabolomics revealed differences in energy metabolism in patients with different subtypes of ischemic stroke [33]. In the present study, 12,13-DHOME was found to be elevated in emphysematous yak lung tissue. While this finding aligns with the hypothesis that altered lipid metabolism may contribute to lung tissue remodeling, further mechanistic investigations are necessary to determine whether 12,13-DHOME actively participates in the emphysematous process or is a secondary consequence of tissue damage.

Asthma pathway, linoleic acid metabolism pathway and gastric acid secretion pathway were common pathways for combined transcriptomic, label-free and untargeted metabolomics analysis. Many lung diseases have tissue remodeling and structural changes, such as emphysema, asthma, chronic obstructive pulmonary disease (COPD) and idiopathic pulmonary fibrosis (IPF) [34]. There was a certain relationship between asthma and lung emphysematous, especially when asthma was not effectively controlled [34]. Transcriptomic comparisons were made between BPD and non-BPD patients, and the study found *CD74*, *HLA-DMA*, *HLA-DRA*, *HLA-DMB*, *HLA-DOB*, *HLA-DQA1*, *HLA-DRB5*, *HLA-DPA1*, *HLA-DOA*, *HLA-DPB1*, *HLA-DQB2*, *HLA-DQA2*, and *HLA-DQB1* were differential genes [35], among which HLA-DQA2 was also the up-regulated expression gene of HLA-DQA2 in the emphysematous lung of yaks, and it was speculated that HLA-DQA2 was also an important signaling molecule affecting the emphysematous process of yaks’ lungs. The integrative analysis identified three common pathways—asthma pathway, linoleic acid metabolism pathway, and gastric acid secretion pathway—across the three omics datasets. While these shared pathways point to coordinated molecular changes linked to emphysema remodeling in yak lung, our current analysis is descriptive and mainly correlative. The limited overlap between bulk-seq and the other two omics layers (only three common pathways) can be explained byseveral factors. First, post-transcriptional and post-translational regulations often decouple mRNAs from protein abundance, as we saw with the discordant expression of BoLA-DQB in this study. Second, differences in analytical sensitivity and dynamic range among the three platforms mean that a pathway may be statistically enriched in one dataset but fall below the detection limit in another. Third, molecular changes in emphysematous remodeling may be compartmentalized, affecting mRNA pools without immediate consequences on protein or metabolite levels, the other way around. Importantly, the three shared pathways (asthma, linoleic acid metabolism, gastric acid secretion) are those that show consistent changes across all molecular layers, which suggests they play central roles in yak lung emphysema. The remaining dataset-specific pathways are not just noise – they reflect the unique biological information captured by each omic approach.

BoLA-DQB is an important immune regulatory gene. In high-throughput sequencing studies of Dabieshan cattle [36], Pinan cattle [37], and kashmir cattle [38], BoLA-DQB was also identified as a key immune response gene. Lundén et al. [39] found that DQA1 haplotypes were significantly linked to susceptibility to clinical mastitis (*p* < 0.05), hinting that the DQB gene may be a candidate gene for mastitis resistance (or susceptibility) in dairy cows and that it plays a vital part in the recovery from inflammatory infections. In this study, BoLA-DQB mRNA was up-regulated while its protein level was down-regulated. The rise of BoLA-DQB mRNA levels went along with the inflammatory state during emphysematous in yak lungs, possibly to help control infectious factors. Meanwhile, the decrease in BoLA-DQB protein levels may be due to various aspects such as post-transcriptional processes, degradation of transcriptional products, translation, and post-translational processing and modification. The observed discordance between BoLA-DQB mRNA and protein expression might be explained by several potential mechanisms: increased protein degradation via the ubiquitin–proteasome system or lysosomal pathways [40], and regulation by non-coding RNAs (e.g., microRNAs) that bind to the 3′UTR of BoLA-DQB mRNA and block translation without affecting mRNA stability [41]. This inconsistency shows why multi-omics integration is valuable: relying solely on transcriptomics would have missed the post-transcriptional regulation of BoLA-DQB. It also highlights underscores the complexity of molecular regulation in emphysematous lung tissue, where mRNA abundance does not always predict protein expression. Future studies combining ribosome profiling, protein turnover assays, and targeted miRNA manipulation are needed to figure out the underlying mechanisms.

We should also note Several limitations, including the relatively small sample size (*n* = 6 per group), lack of functional validation, and cross-sectional design. Individual sex and exact body weight were not recorded, but any leftover confounding is probably minimal. Histopathological assessment was qualitative, since the primary aim was binary classification. A post hoc power analysis showed that the study has reasonably powered to detect major molecular changes (median power: 0.86 for transcriptomics, 0.79 for proteomics, 0.74 for metabolomics). Nevertheless, validation in larger independent groups and functional experiments are needed. We also acknowledge the lack of independent validation using qPCR, Western blotting, or targeted metabolomics. Future studies will prioritize such validations and also use cross-omics correlation modeling (e.g., WGCNA, SNF) to better understand regulatory modules in yak high-altitude pulmonary emphysema.

Transcriptomics, label-free techniques and untargeted metabolomics complement one another in the study of lung emphysema, and using them together can provide us with a fuller and deeper understanding of the molecular mechanisms behind lung emphysema. This study points out the direction and priorities for exploring the molecular mechanisms of yak lung emphysema, offers omics data for studying yak plateau adaptation at the molecular level, and provides a theoretical basis for building a prognostic evaluation system of lung emphysema.

## 5. Conclusions

In this study, transcriptomic, proteomic, and non-targeted metabolomic analyses were conducted on both normal and emphysematous yak lung tissues. The investigation identified 254 differentially expressed genes, 554 differentially expressed proteins, and 21 distinct metabolites. These comprehensive analyses primarily focused on the asthma pathway, the linoleic acid metabolism pathway, and the gastric acid secretion pathway. These findings provide preliminary descriptive insights into emphysematous remodeling in yak lung and should be considered hypothesis-generating.

## Figures and Tables

**Table 1:** The number of differentially expressed genes in transcriptomics.

Control	Treat	Up-Regulated	Down-Regulated	Total
CG	EG	174	80	254

**Table 2:** The number of differentially expressed proteins in label-free.

Control	Treat	Up-Regulated	Down-Regulated	Total
CG	EG	299	255	554

## Data Availability

The RNA-sequencing data have been deposited in the Sequence Read Archive under BioProject accession number PRJNA1184027. The proteomic data are available in the iProX database under Project accession number IPX0010196000. The metabolomics data are accessible in the Metabolomics under Project accession number MTBLS11566.

## Supplementary Materials

The following supporting information can be downloaded at: https://www.mdpi.com/article/10.3390/ani16121775/s1. Supplementary Figure S1 presents histological observation of yak lung tissues; Supplementary Figure S2 shows GO enrichment analysis of differentially expressed genes; Supplementary Figure S3: Principal component analysis (PCA) of transcriptomic data from normal and emphysematous yak lungs; Supplementary Figure S4: Principal component analysis (PCA) of label-free proteomic data from normal (CG, *n* = 6) and emphysematous (EG, *n* = 6) yak lungs; Supplementary Figure S5 presents PLS-DA analysis of untargeted metabolomics data, including (A) positive ion mode score plot, (B) permutation test for positive ion mode, (C) negative ion mode score plot, (D) permutation test for negative ion mode; Supplementary Figure S6 shows a volcano plot of differential metabolites in untargeted metabolomics. Supplementary Table S1 reports the median post hoc statistical power values for each omics dataset (transcriptomics, proteomics, and metabolomics).
