# Supplementary material for: Multi-Omics Analysis Reveals New Insights into Yak Lung Under High-Altitude Adaptation
Source: Animals (Basel). 2026 Jun 8;16(12):1775. doi: 10.3390/ani16121775 (PMC13295351; doi:10.3390/ani16121775)
Supplement: Supplementary file 1 [file animals-16-01775-s001.zip › Supplementary Table S1.docx]

Supplementary Table S1. Post hoc statistical power for each omics dataset.

| Omics dataset | Number of significant molecules | Median power | Percentage of significant molecules with power > 0.8 |
| --- | --- | --- | --- |
| Transcriptomics | 254 (174 up,  80 down) | 0.86 | 82% |
| Label‑free proteomics | 554 (299 up,  255 down) | 0.79 | 71% |
| Untargeted metabolomics | 21 (12 up,  9 down) | 0.74 | 67% |

**Note:** Power was calculated using observed effect sizes (log₂FC) and variance estimates, assuming a two‑sided t‑test with α = 0.05.
