# Supplementary figures and images for: Multi-Omics Analysis Reveals New Insights into Yak Lung Under High-Altitude Adaptation
Source: Animals (Basel). 2026 Jun 8;16(12):1775. doi: 10.3390/ani16121775 (PMC13295351; doi:10.3390/ani16121775)

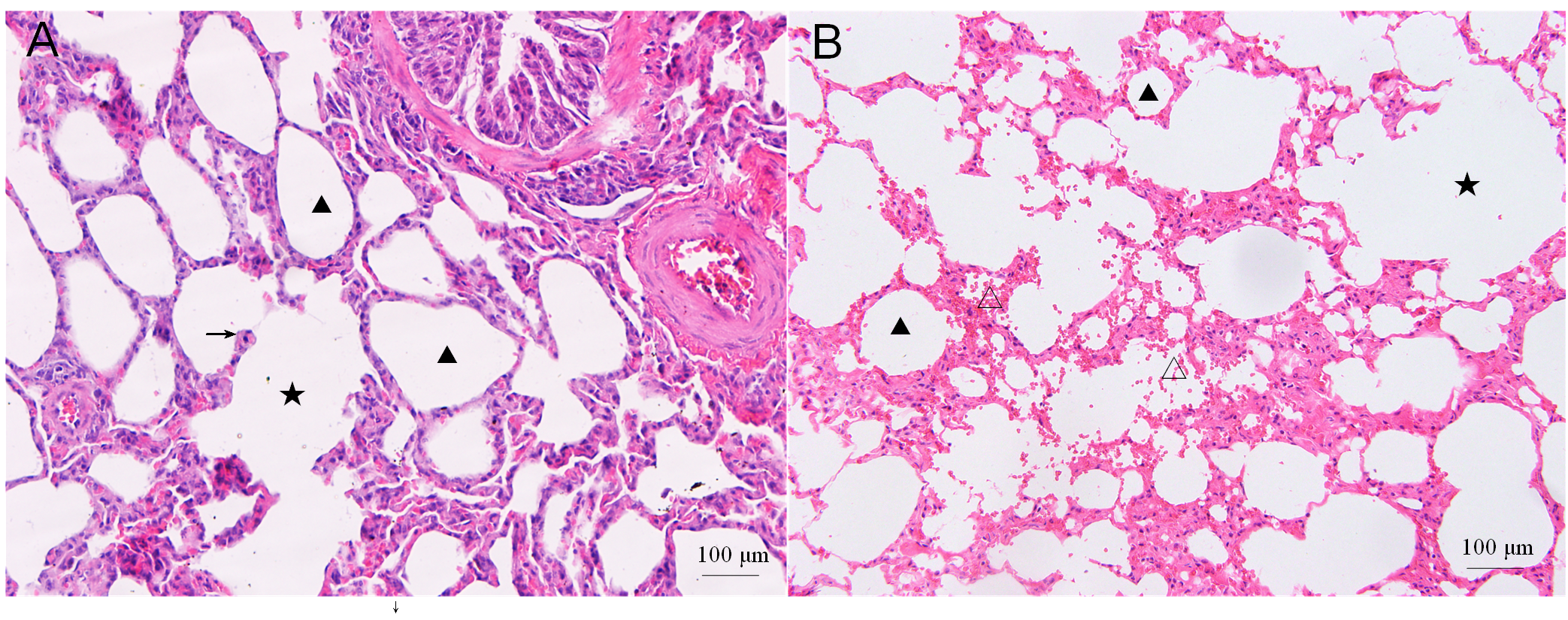

Supplement: Supplementary file 1 [file animals-16-01775-s001.zip › Supplementary Figure S1.tif]

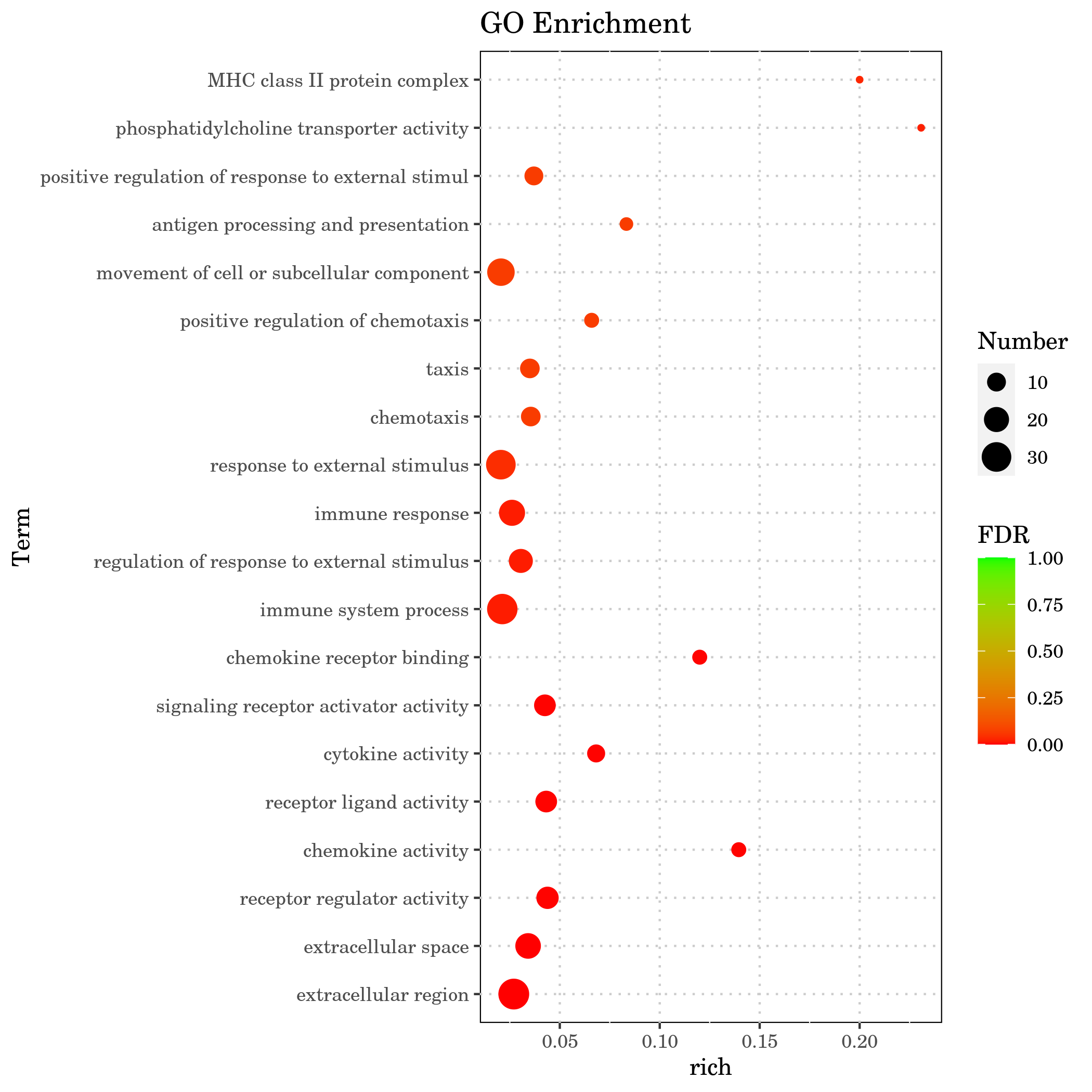

Supplement: Supplementary file 1 [file animals-16-01775-s001.zip › Supplementary Figure S2.tif]

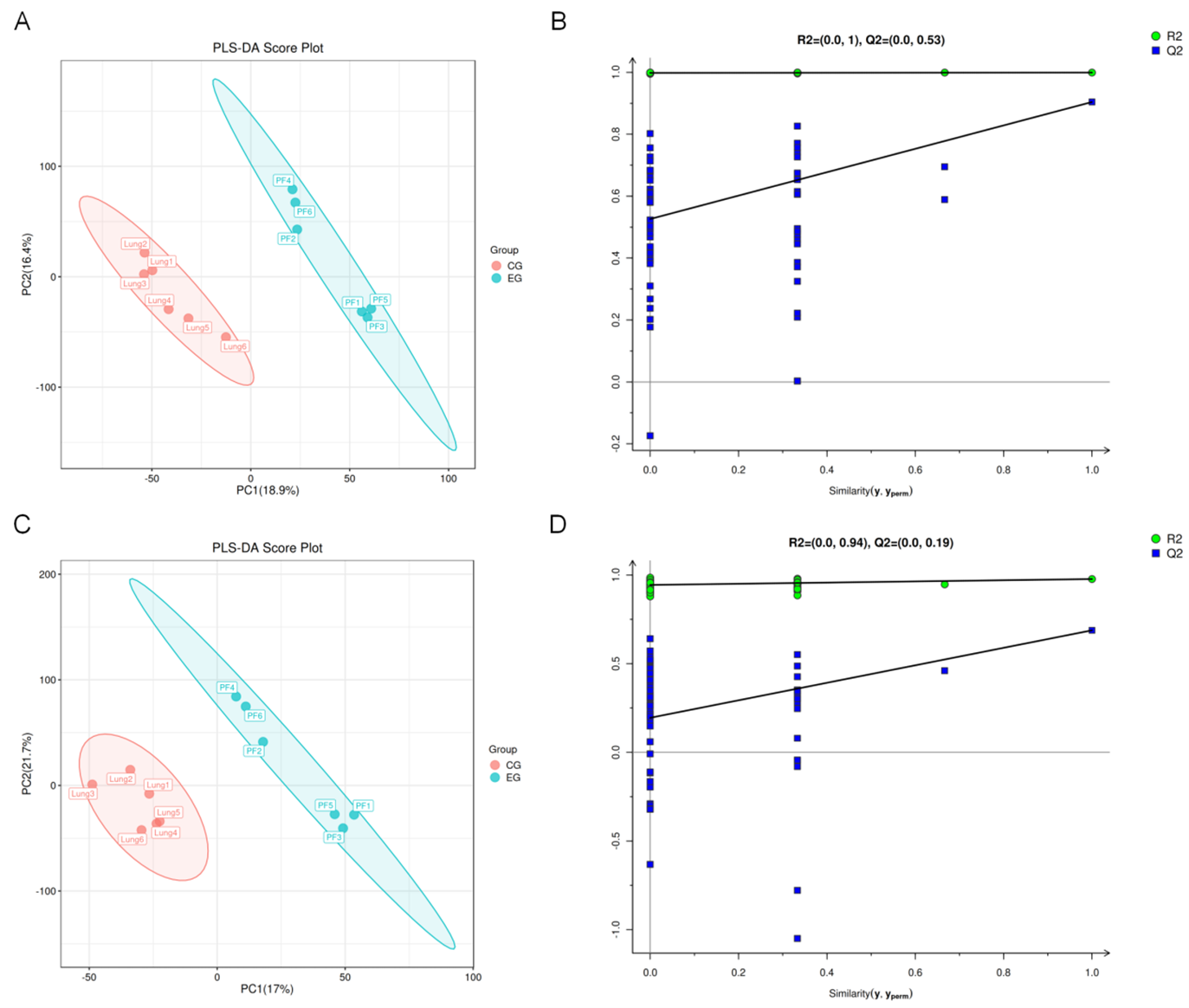

Supplement: Supplementary file 1 [file animals-16-01775-s001.zip › Supplementary Figure S3.tif]

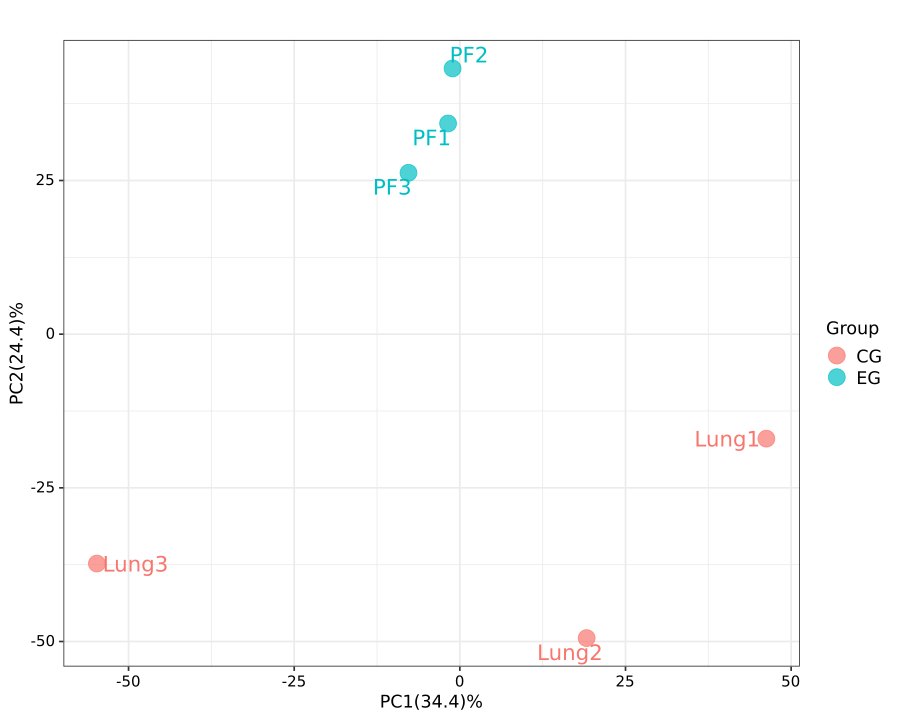

Supplement: Supplementary file 1 [file animals-16-01775-s001.zip › Supplementary Figure S4.png]

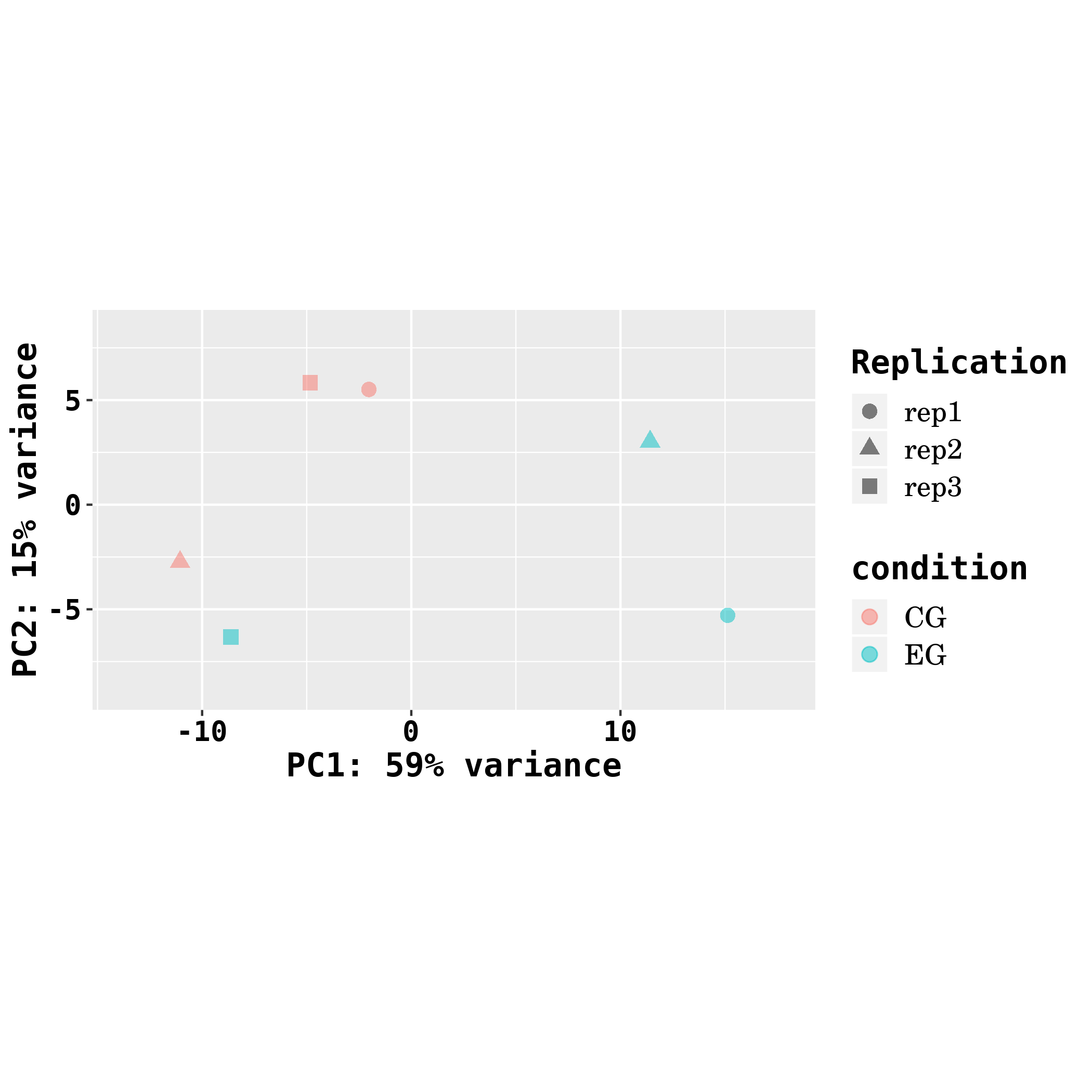

Supplement: Supplementary file 1 [file animals-16-01775-s001.zip › Supplementary Figure S5.png]

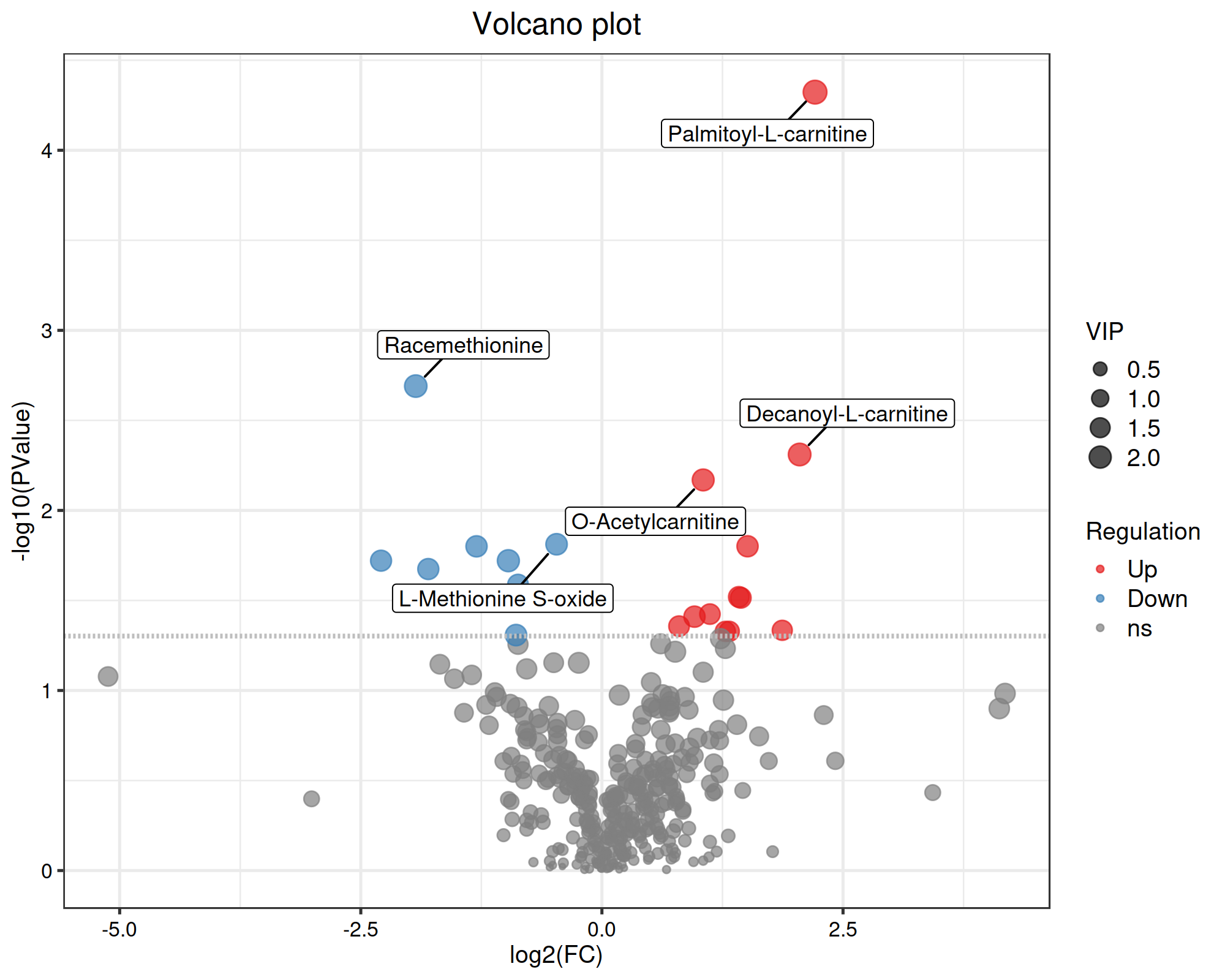

Supplement: Supplementary file 1 [file animals-16-01775-s001.zip › Supplementary Figure S6.png]
